# Asynchronous Bilateral Ureteric-Arterial Fistula: Diagnosis and Treatment

**DOI:** 10.1155/2021/5590432

**Published:** 2021-05-03

**Authors:** Pietro Pepe, Letterio D'Arrigo, Domenico Patane', Ludovica Pepe, Giuseppe Candiano, Michele Pennisi

**Affiliations:** ^1^Urology Unit-Cannizzaro Hospital, via Messina 829, Catania, Italy; ^2^Imaging Department-Cannizzaro Hospital, Catania, Italy

## Abstract

A 48-year-old woman submitted to anterior exenteration plus ileal-cutaneous conduit for metastatic cervical cancer during the change of the ureteral stent showed massive bleeding in the left ureter. A selective intra-arterial angiography showed a fistula between the ureter and the left common iliac artery that the interventional radiologist quickly repaired by inserting a vascular endoprosthesis. Six months later, gross hematuria secondary to right ureter-iliac fistula occurred again and a second endoprosthesis was inserted. Asynchronous bilateral ureteric stent-related vascular fistula is an uncommon scenario, but it should be suspected in the presence of hematuria following ureteral stent replacement.

## 1. Introduction

The ureteric-arterial fistula (UAF) is a rare condition characterized by a direct fistulous communication between the ureter and the iliac artery resulting in bleeding into the ureter that was described for the first time by Moschcowitz in 1908 [1]. The UAF frequently can be associated with previous vascular and oncological surgery (bladder, gynecological, or colorectal cancer), aneurysm, or pseudoaneurysm of the common or external iliac artery, radiotherapy, and previous ureteral damage or alteration of the urinary system (stenosis, urinary diversion, or ureteral stent placement). Since these bleedings occur intermittently, the diagnosis is very difficult; in fact, UAF can be life-threatening because of potential massive blood loss, and diagnosis can easily be delayed or even missed because the presence of hematuria could be ascribed to variety of other urologic diseases. The mortality associated with UAF ranges from 7 to 58%, and the clinical outcome is correlated with an early diagnosis [2, 3]. UAF requires a rapid a multidisciplinary approach, including the expertise of the urologist, vascular surgeon, and interventional radiologist, to detect and treat this unusual disease [4, 5].

In this study, we report the clinical management of non-synchronous bilateral ureteric-iliac fistula.

## 2. Case Presentation

In 2009, a 48-year-old woman underwent radical hysterectomy for cervical cancer followed by adjuvant radiotherapy and chemotherapy. After two years, the patient was submitted to ureter-ileal-cutaneous conduit because of pelvic metastatic disease. During a check-up two years from anterior exenteration, a fibrosis plate in the lower pelvis that compressed both ureters and subsequently caused a hydronephrosis on both sides was diagnosed by abdominal computed tomography (CT). Therefore, at first, the kidneys were drained by percutaneous nephrostomy those later replaced by bilateral ureteral stent.

**Figure 1:**
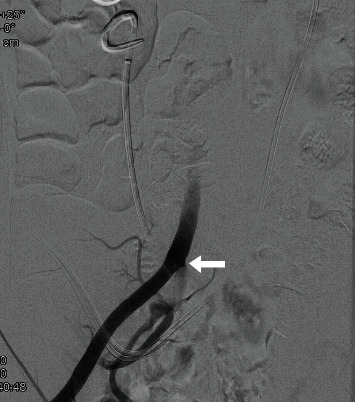
Intra-arterial angiography in digital technique showed a fistula between the common iliac artery and the ureter.

**Figure 2:**
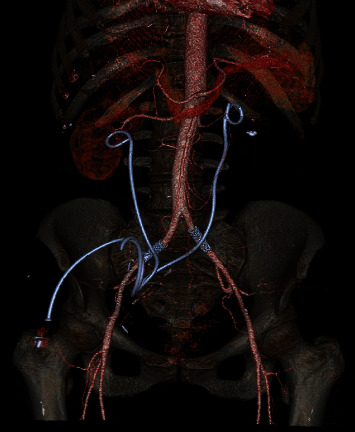
Three-dimensional abdominal CT scan that demonstrated bilateral iliac artery endoprosthesis and ureteral stents (axial evaluation).

**Figure 3:**
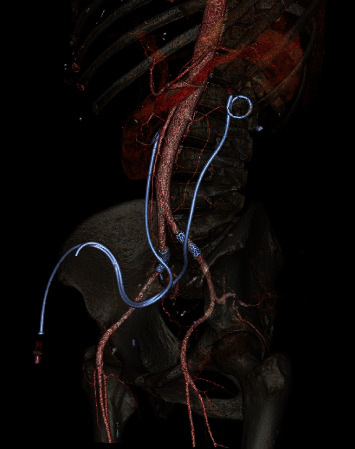
Three-dimensional abdominal CT scan that demonstrated bilateral iliac artery endoprosthesis and ureteral stents (sagittal evaluation).

During a programmed change of the ureteral stent in September 2019, a massive bleeding in the left ureter was showed; through the quick change of the stent, a spontaneous tamponade was made, and after stabilizing the cardiovascular system and correcting the heavy loss of blood, a CT scan followed by a selective intra-arterial angiography in digital technique was carried out. Both examinations showed an ureteroarterial fistula between the left common iliac artery and the ureter (Figure 1). During the examination, the interventional radiologist successfully repaired the common and the left common iliac artery by inserting a vascular endoprosthesis (stent Bentley BeGraft 10/37 mm). The patient was discharged after five days following CT scan. Six months later, hematuria with anemia occurred again after the programmed bilateral change of the ureteral stent; the patient quickly underwent CT-angiogram that showed the presence of an ureteroiliac fistula of the right side that was repaired by inserting a second endoprosthesis (Figures 2 and 3). The patient was successfully discharged after four day and still today is asymptomatic.

## 3. Discussion

Ureteral stent-related UAF are reported in a small number of cases; the causative factor has been attributed to prolonged stent placement varying from 5 months to 15 years before the onset of hematuria [2]. The etiology of UAF is not well understood; a possible mechanism is related to the alteration in the ureteral wall elasticity with eventual pressure necrosis [3]. The other plausible theory is postoperative inflammatory reaction or associated infection leading to inflammatory ureteral fixation to the wall of the arteria leading to erosion. In the majority of ureteric-iliac fistula, the mechanical forces due to the pulsatility of the common iliac artery against the ureter that contribute to the erosion are predisposed by underlying vascular pathology, previous radiation therapy, and previous surgery, including genitourinary, pelvic, vascular operations, or the need of long-term ureteral stents. The hematuria is either intermittent or life-threatening, with some patients presenting spontaneously and others during stent exchange. In fact, most of the patients with a diagnosis of UAF have a history of pelvic malignancy; in addition, the increasing incidence in last decades is related to the increased number of various treatment introduced in clinical practice that predispose the development of a weakness tissue condition. The diagnosis of ureteric-arterial fistula could be elusive and may be delayed depending on the clinical scenario. The sensitivity of the standard angiography examination is 23-41%; but it can be improved to 63% using the “provocative” method, which means mobilizing the ureteral stent during examination. Other diagnostic studies, such as retrograde pyelogram and ureteroscopy, add minimal value in the presence of significant bleeding other than lateralizing the source of the hematuria. Almost 90% of patients with a correct diagnosis are treated successfully; on the other hand, the mortality for patients with an undiagnosed condition is up to 58%.

Multiple treatment options, such as open repair with ligation, embolization of the hypogastric artery or endovascular stenting, are available with various success rates; endovascular stenting has been proven to be effective and provides a faster resolution especially in a setting of massive hemorrhage [4, 5]. To our knowledge, about 140 cases have been described in the literature, but only one paper refers to bilateral ureteric-iliac fistula repair [3]; the mortality rate through UAF has decreased from 69% in 1980 to 7-23% today.

In our case, the patient presented with intermittent hematuria following the ureteral stent replacement; although the initial diagnosis of left ureteric-iliac fistula was delayed because the unusual clinical presentation, conversely, the presence of recurrent hematuria allowed to quickly diagnose the contralateral ureteroiliac fistula that was restored by an interventional radiologist.

## 4. Conclusion

Ureteric stent-related vascular fistula is an uncommon scenario, but it should be suspected in the presence of early and/or delayed hematuria following ureteral stent replacement; in addition, a multidisciplinary approach (interventional radiologist, vascular surgeon and urologist) is mandatory to achieve the best clinical results.
